# A novel approach in the treatment of neuroendocrine gastrointestinal tumours. Targeting the epidermal growth factor receptor by gefitinib (ZD1839)

**DOI:** 10.1038/sj.bjc.6601346

**Published:** 2003-10-28

**Authors:** M Höpfner, A P Sutter, B Gerst, M Zeitz, H Scherübl

**Affiliations:** 1 Medical Clinic I, Gastroenterology/Infectious Diseases/Rheumatology, University Hospital Benjamin Franklin, Free University of Berlin, Hindenburgdamm 30, 12200 Berlin, Germany

**Keywords:** NE tumours, apoptosis, cell-cycle, EGFR, TK

## Abstract

Therapeutic options to inhibit the growth and spread of neuroendocrine (NE) gastrointestinal tumours are still limited. Since gefitinib (4-(3-chloro-4-fluoroanilino)-7-methoxy-6-(3-morpholinopropoxy)quinazoline), an inhibitor of epidermal growth factor receptor-sensitive tyrosine kinase (EGFR-TK), had been shown to suppress potently the growth of various non-NE tumour entities, we studied the antineoplastic potency of gefitinib in NE gastrointestinal tumour cells. In human insulinoma (CM) cells, in human pancreatic carcinoid (BON) cells and in NE tumour cells of the gut (STC-1), gefitinib induced a time- and dose-dependent growth inhibition by almost 100%. The antiproliferative potency of gefitinib correlated with the proliferation rate of the tumour cells. So the IC_50_ value of gefitinib was 4.7±0.6 *μ*M in the fast-growing CM cells, still 16.8±0.4 *μ*M in the moderate-growing BON cells, and up to 31.5±2.5 *μ*M in the slow-growing STC-1 cells. Similarly, the induction of apoptosis and cell-cycle arrest by gefitinib differed according to growth characteristics: fast-growing CM cells displayed a strong G0/G1 arrest in response to gefitinib, while no significant cell-cycle alterations were seen in the slow-growing STC-1. *Vice versa*, the proapoptotic effects of gefitinib, as determined by caspase-3 activation and DNA fragmentation, were most pronounced in the slow-growing STC-1 cells. Using cDNA microarrays, we found extensive changes in the expression of genes involved in the regulation of apoptosis and cell cycle after incubation with gefitinib. Among them, an upregulation of the growth arrest and DNA damage-inducible gene GADD153 was observed. Phosphorylation of ERK1/2, which inhibits GADD153 expression, was reduced in a time-dependent manner. However, no gefitinib-induced activation of the GADD153-inducing p38 mitogen-activated protein kinase was detected. Our data demonstrate that the inhibition of EGFR-TK by gefitinib induces growth inhibition, apoptosis and cell-cycle arrest in NE gastrointestinal tumour cells. Thus, EGFR-TK inhibition appears to be a promising novel approach for the treatment of NE tumour disease.

Gastrointestinal neuroendocrine (NE) tumours represent a rare and rather heterogeneous tumour entity. Almost half of the metastatic NE gastrointestinal tumours release excessive amounts of biogenic amines and/or neuropeptides, thereby causing a characteristic hypersecretion syndrome. The often bizarre clinical symptoms are generally well controlled by somatostatin analogues or interferon-*α* (Faiss *et al*, 1996; Öberg, 2001). Therapeutic options to inhibit growth and further spread of metastatic NE tumours are yet very limited. Conventional chemotherapy is indicated in patients with anaplastic and fast-growing NE tumours, but provides no survival benefit for patients with slow disease progression. In the light of the particular biology of NE tumour disease, innovative treatment strategies should be developed that are both effective and well tolerated.

Recently, evidence has been accumulated indicating that the epidermal growth factor receptor (EGFR) is a promising target for novel cancer therapy. A great variety of tumours show abnormal, enhanced and/or constitutive expression of EGFR. Moreover, EGFR (over-) expression has been correlated with disease stage, reduced survival, development of tumour metastases and tumour differentiation in various cancers (Baselga, 2002; Di Lorenzo *et al*, 2002; Peghini *et al*, 2002). Several reports indicate that EGFR are expressed frequently in NE tumours in general (Wang *et al*, 1997; Ezzat, 2001), and in gastrointestinal NE tumours in particular (Nilsson *et al*, 1993, 1995; Öberg, 1994; Shimizu *et al*, 2000; Peghini *et al*, 2002). In addition, EGFR contributes to the growth characteristics of NE tumours (Nilsson *et al*, 1995; Wulbrand *et al*, 1998; Peghini *et al*, 2002). Hence, EGFR is an attractive target for innovative treatment strategies in gastrointestinal NE tumour disease.

The EGFR is part of a subfamily of four closely related receptors: EGFR (ErbB-1), HER-2/*neu* (ErbB-2), HER-3 (ErbB-3) and HER-4 (ErbB-4). Upon ligand binding EGFR becomes activated by dimerisation. The receptor activation leads to the subsequent activation of EGFR tyrosine kinase (TK) enzymatic activity, which plays a central role in receptor-mediated signal transduction, cell mitogenesis and cell transformation (Baselga, 2002). Inhibiting EGFR and their specific TK activity is regarded as a very promising approach for innovative therapeutic strategies in cancer treatment (Arteaga, 2002).

Gefitinib (4-(3-chloro-4-fluoroanilino)-7-methoxy-6-(3-morpholinopropoxy)quinazoline (ZD1839)), a specific EGFR-TK inhibitor, is currently in clinical testing for various tumour entities (Baselga *et al*, 2002; Herbst, 2002). Gefitinib is a low molecular weight (MW: 447), synthetic anilinoquinazoline. The orally available and reversibly acting drug is highly specific for EGFR-TK, exhibiting almost no activity against other TKs and several serine/threonine kinases (Woodburn *et al*, 1998; Ciardiello and Tortora, 2001). Antineoplastic properties of gefitinib have been demonstrated in a wide range of human cancers, including prostate, breast, ovarian, colon, epidermoid and lung cancer cells (Ciardiello *et al*, 2000; Cullinane *et al*, 2000; Sirotnak *et al*, 2000; Chan *et al*, 2001; Di Lorenzo *et al*, 2002; Sewell *et al*, 2002).

EGFR-TK inhibition has not yet been evaluated in the antineoplastic treatment of NE tumours. Hence, in the present study, we examined the antineoplastic potency of the selective EGFR-TK inhibitor gefitinib in a set of NE gastrointestinal tumour cell lines with different growth characteristics. We focused on gefitinib-induced growth inhibition, its induction of apoptosis and regulation of the cell cycle in NE gastrointestinal tumour cells.

## MATERIAL AND METHODS

### Cell lines

Human pancreatic carcinoid BON cells (Ahnert-Hilger *et al*, 1996; Lemmer *et al*, 2002) were maintained in a 1 : 1 mixture of DMEM, and F12K medium containing 10% FCS (Biochrom, Berlin, Germany) and 1% L-glutamine. The human insulinoma cell line CM (Baroni *et al*, 1999), kindly provided by Professor P Pozzilli (University La Sapienza of Rome, Italy), was cultured in RPMI 1640 supplemented with 5% FCS (Biochrom) and 1% L-glutamine. The murine intestinal NE tumour cell line STC-1 (Höpfner *et al*, 2002), which was a gift from Dr D Hanahan (University of California, San Francisco, CA, USA), was maintained in DMEM supplemented with 15% horse serum (Biochrom), 2.5% FCS (Biochrom) and 1% L-glutamine. All cell lines were kept at 37°C in a humidified atmosphere (5% CO_2_).

### Drugs

Gefitinib was a kind gift from AstraZeneca, Great Britain. Nonradiolabelled meta-iodobenzylguanidine (MIBG) was kindly provided by Amersham Buchler (Braunschweig, Germany). Stock solutions were prepared in DMSO and stored at −20°C. The drugs were diluted in fresh media before each experiment. In all experiments, the final DMSO concentration was <0.5%. To evaluate the effects of gefitinib and/or meta-iodobenzylguanidine, cells were incubated with either control medium or a medium containing rising concentrations of the respective drug or drug combination. Media were changed daily to ensure constant drug concentrations in the incubation medium.

### Reverse transcriptase–polymerase chain reaction (RT–PCR)

The total RNA was extracted from cultured cell lines with RNAClean, following the recommendation of the manufacturer (Hybaid, London, UK). Reverse transcription and PCR reactions were carried out as described elsewhere in detail (Glassmeier *et al*, 1998). To eliminate any possible contamination with genomic DNA, RNAs were treated with 1 U DNAse I (Gibco, Karlsruhe, Germany) per *μ*g RNA for 15 min at room temperature. The possible contamination with genomic DNA was excluded by control experiments omitting the reverse transcriptase. Purified RNA was reverse transcribed into cDNA using oligo-dT-primers and the SuperScript Preamplification-Kit (Gibco). PCR reactions were carried out in a total volume of 50 *μ*l containing 400 nM of each primer, 200 *μ*M of each dNTP (Pharmacia, Uppsala, Sweden), 50 mM KCl, 1.5 mM MgCl_2_, 10 mM tris(hydroxymethyl)-aminomethane (Tris) and 1 U *Taq*-Polymerase (Pharmacia). PCR was performed in a Peltier thermocycler (PTC-200, MJ Research, Watertown, MA, USA) with primers and conditions as indicated in Table 1

**Table 1:** Primer sequences and PCR conditions for the detection of mRNA expression of the indicated genes in NE gastrointestinal tumour cells

**Genes**	**Primers (5′–3′)**	**Position in the mRNA**	**Product size (bp)**	**Denaturating temperature and time (s)**	**Annealing temperature and time (s)**	**Extension temperature and time (s)**	**Number of cycles**
EGFR-1	F: TCCTCCCAGTGCCTGAATAC	3442–3462	240	94°C (40)	63°C (60)	72°C (60)	30
	R: TAATTTGGTGGCTGCCTTTC	3682–3663					
EGFRvIII^a^	F: GGCTCTGGAGGAAAAGAAAG	255–274	226	94°C (40)	63°C (60)	72°C (60)	30
	R: TGATGGAGGTGCAGTTTTTG	1282–1263					
IGRR-*β*1	F: GAAGTGGAACCCTCCCTCTC	1932–1951	241	94°C (40)	63°C (60)	72°C (60)	30
	R: GTTCTCGGCTTCAGTTTTGG	2172–2153					
*β*-actin	F: ATCATGTTTGAGACCTTCAACAC	437–459	822	94°C (40)	63°C (60)	72°C (60)	30
	R: TCTGCGCAAGTTAGGTTTTGTC	1237–1258					

a EGFRvIII does not possess an mRNA-sequence distinct from the EGFR-1 sequence, but Exons 2–7 are missing. Using the indicated primers, EGFRvIII is characterised by a product of 226 bp size, while the wild-type receptor is recognised with a bp product of 1028 bp.

All templates were initially denaturated for 5 min at 95°C and the amplification was extended at a final extension temperature of 72°C for 7 min.

.

### cDNA array

For determination of gefitinib-induced differential gene expression, human CM insulinoma cells were treated with gefitinib (10 *μ*M) for 48 h. Untreated cells served as controls. Isolation of the total RNA of treated and untreated cells was performed as described above. Polyadenylated (polyA^+^) mRNAs were enriched using magnetic Dynabeads, according to the instructions of the supplier (Dynal, Oslo, Norway). The quality of total and polyA^+^ RNA was controlled by agarose gel electrophoresis. Labelled first-strand cDNA probes were prepared from the polyA^+^ RNAs of both gefitinib-treated and control samples. Each sample was hybridised to an individual membrane of identically spotted 205 apoptosis- and cell-cycle-related genes (Human Apoptosis Array; Clontech, Palo Alto, CA, USA). A complete list of the cDNAs and controls as well as their accession numbers is available on the web (http://atlasinfo.clontech.com /genelists/huApop.xls). After washing, according to the manufacturer's instructions, the membranes were exposed to an X-ray film for quantification. Alteration in the expression of a respective gene is given as fold increase/or decrease compared with the signal of the untreated control (Höpfner *et al*, 2002). The hybridisation signals were photometrically evaluated using TINA software (Raytest Isotopenmessgeräte, Straubenhardt, Germany). For determination of up- and downregulation, the mean optical density/mm^2^ (OD/mm^2^) of each gene was identified and normalised to housekeeping gene expression (ubiquitin, GAPDH, tubulin *α* 1 subunit, HLAC, cytoplasmic *β*-actin, 60S ribosomal protein L13A, 40S ribosomal protein S9). Then the ratio of gene expression in treated *vs* untreated cells was calculated. The cutoff for upregulation was set at a 1.5-fold increase of the OD/mm^2^ ratio of genes in the treated samples, whereas downregulation was determined as the 0.67-fold OD/mm^2^ (reciprocal value of 1.5-fold increase) expression of genes in the treated samples.

### Western blotting

Whole-cell extracts were prepared by harvesting and lysing cells with lysis buffer (sodium dodecyl sulphate (SDS) 0.1%, sodium deoxycholic acid 0.5%, Nonidet P-40 1%, phenylmethylsulphonyl fluoride (PMSF) 0.1 mM, aprotinin 1 *μ*g ml^−1^, pepstatin A 1 *μ*g ml^−1^). The protein content of the lysate was determined using the BCA protein assay kit (Pierce, Rockford, IL, USA). The cell lysate was mixed with gel-loading buffer (Tris-HCl 62.5 mM, glycerol 10%, SDS 1%, *β*-mercaptoethanol 2.5%). After boiling for 5 min, the lysates were subjected to SDS/polyacrylamide gel electrophoresis (30 *μ*g of protein per lane; gel: polyacrylamide 10%, SDS 0.1%, Tris-HCl 25 mM; running buffer: Tris 25 mM, glycine 50 mM, 0.1% SDS). After electrophoresis, gels were equilibrated with transfer buffer (Tris-HCl 25 mM, glycine 50 mM, 20% methanol). Proteins were transferred onto nitrocellulose membranes by electroblotting (BioRad, Munich, Germany). Blots were blocked in 1.5% bovine serum albumine (BSA), and then incubated at 4°C overnight with the total p38 mitogen-activated protein kinase (MAPK) and phospho-p38 MAPK or the total extracellular signal-regulated kinase 1/2 (ERK1/2) and phospho-ERK1/2 antibodies, respectively (1 : 500, Santa Cruz Biotechnology, CA, USA). After washing with phosphate-buffered NaCl solution (PBS) containing 0.1% Tween and incubation with horseradish peroxidase-coupled secondary antibody (1 : 10 000, Amersham, Uppsala, Sweden) at room temperature for 1 h, the blot was washed extensively and developed using enhanced chemiluminescent detection (Amersham, Uppsala, Sweden). Blots were exposed to Hyperfilm ECL film (Amersham, Uppsala, Sweden) for 1–5 min.

### Immunofluorescence labelling

Cells were trypsinised, washed twice with PBS and immunostained, as described previously (Sutter *et al*, 2002). Then samples were fixed and permeabilised using the Fix & Perm cell permeabilisation kit (Caltag, Laboratories, Hamburg, Germany). Cells were incubated for 1 h at room temperature with the primary rabbit polyclonal IGF-*β*1 receptor antibody (5 *μ*g ml^−1^, # sc-713, Santa Cruz) or with rabbit polyclonal EGF receptor antibody (5 *μ*g ml^−1^, # sc-03, Santa Cruz), both mapping at the respective C-terminus of the protein. Negative controls were performed by omitting the primary antibody. Cells were washed twice with PBS and then incubated with secondary FITC-labelled polyclonal anti-rabbit Ig antibody (5 *μ*g ml^−1^, BD Pharmingen, Heidelberg, Germany) for 1 h at room temperature. Fluorescence was detected by flow cytometry on a FACSCalibur (Becton Dickinson, Heidelberg, Germany) and analysed using CellQuest software.

### Measurement of growth inhibition

Changes in the cell number of BON, CM and STC-1 cells were determined by crystal violet staining after 96 h of incubation with rising concentrations of gefitinib (0–50 *μ*M). Measurements were performed as described (Höpfner *et al*, 2001). Cells were washed with PBS and fixed with 1% glutaraldehyde. After another washing step, cells were stained with 0.1% crystal violet. The unbound dye was removed by washing. Crystal violet that had absorbed onto the cells was solubilised with 0.2% Triton X-100. Then light extinction was analysed at 570 nm using an ELISA reader.

### Caspase-3 activity assay

Preparation of cell lysates and determination of caspase-3 activity was performed as described previously (Maaser *et al*, 2002). The activity of caspase-3 was calculated from the cleavage of the fluorogenic substrate DEVD-AMC (Calbiochem-Novabiochem, Bad Soden, Germany). Cell lysates were incubated with substrate solution (caspase-3 substrate AC-DEVD-AMC 20 *μ*g ml^−1^, HEPES 20 mM, glycerol 10%, DTT 2 mM, pH 7.5) for 1 h at 37°C, and the cleavage of DEVD-AMC was measured fluorometrically with a VersaFluor fluorometer (excitation: 360 nm emission: 460 nm) from Biorad, Munich, Germany.

### DNA fragmentation

DNA fragmentation was determined by performing Cell Death Detection ELISA (Roche) as described previously (Höpfner *et al*, 2003). Briefly, after 48 h of incubation, cells were lysed with incubation buffer. The cytoplasmic fractions were diluted to contain 2.5 × 10^3^ cell equivalents per ml, and the presence of mono- and oligonucleosomes was assayed using antibodies directed against DNA and histones. DNA fragments were detected by a peroxidase system with colour development read at 405 nm.

### Cell-cycle analysis

Cell-cycle analysis was performed by the method of Vindelov and Christensen, as described previously (Maaser *et al*, 2001). Cells were trypsinised, washed and the nuclei were isolated using the CycleTest PLUS DNA Reagent Kit (Becton Dickinson, Heidelberg, Germany). DNA was stained with propidium iodide according to the manufacturers' instructions. The DNA content of the nuclei was detected by flow cytometry and analysed using CellFit software (Becton Dickinson).

### Statistical analysis

The antineoplastic effects of the various substances and vehicles were compared by the unpaired, two-tailed Mann–Whitney *U*-test. The unpaired Student's *t*-test was used for cell-cycle analysis. *P*-values were considered to be significant at <0.05. If not stated otherwise, all functional experiments were performed in quadruplicate.

## RESULTS

### Expression of EGFR and IGFR in NE gastrointestinal tumour cells

**Figure 1:**
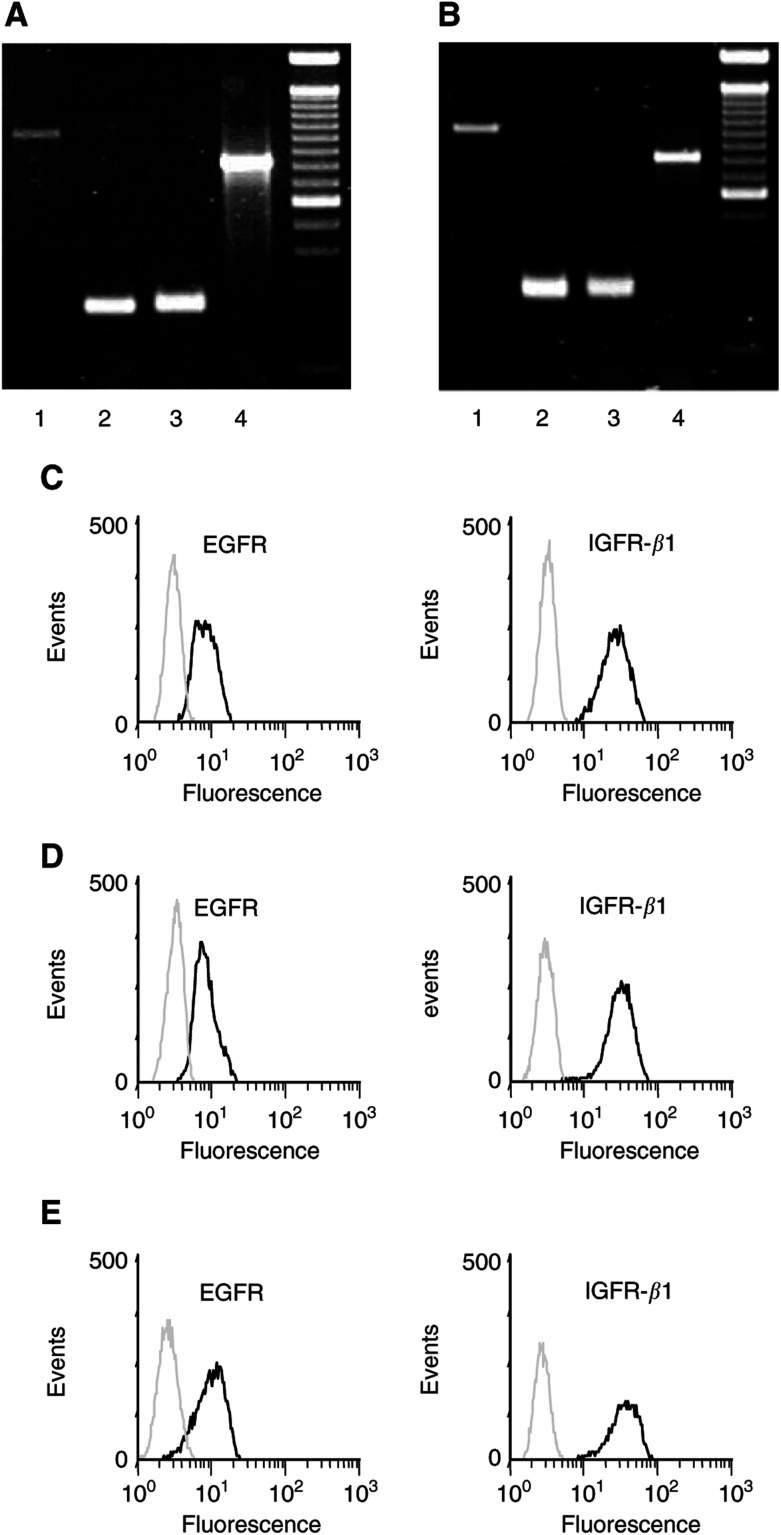
mRNA and protein expression of EGFR and IGFR in neuroendocrine tumour cells. (**A**, **B**) mRNA expression of EGFRvIII (lane 1), EGFR-1 (lane 2) and IGFR*β*-1 (lane 3) was evaluated in CM (**A**) and BON tumour cells (**B**). *β*-Actin was used as positive control (lane 4 in **A** and **B**), 100 bp DNA ladder. (**C**–**E**) Flow cytometric analysis of the expression of EGFR and IGFR*β*-1 proteins in CM cells (**C**), BON cells (**D**) and STC-1 cells (**E**). Black lines: cells stained with specific polyclonal antibodies against either EGFR or IGFR*β*-1; grey lines: negative controls.

The mRNA expression of EGF receptors (EGFR) and the insulin-like growth factor receptor *β*-1 (IGFR-*β*1) was investigated in human BON and in human CM cells. The mRNAs specific for EGFR and IGFR*β*-1 were detected in both the cell lines (Figure 1A, B

). To evaluate protein expression of both EGFR and IGFR*β*-1, cells were stained with specific antibodies and analysed by flow cytometry. Protein expression of EGFR and IGFR-*β*1 was detected in both the cell lines (Figure 1C, D). By contrast, no expression of the EGFR mutation, EGFRvIII, often observed in non-NE cancer types, was detected in the human NE tumour models used (Figure 1A, B). Labelling murine STC-1 cells with specific antibodies for EGFR and IGFR-*β*1 also confirmed the expression of both growth factor receptors in this model of NE gut tumour cells (Figure 1D).

### Growth-inhibitory effects of gefitinib

**Figure 2:**
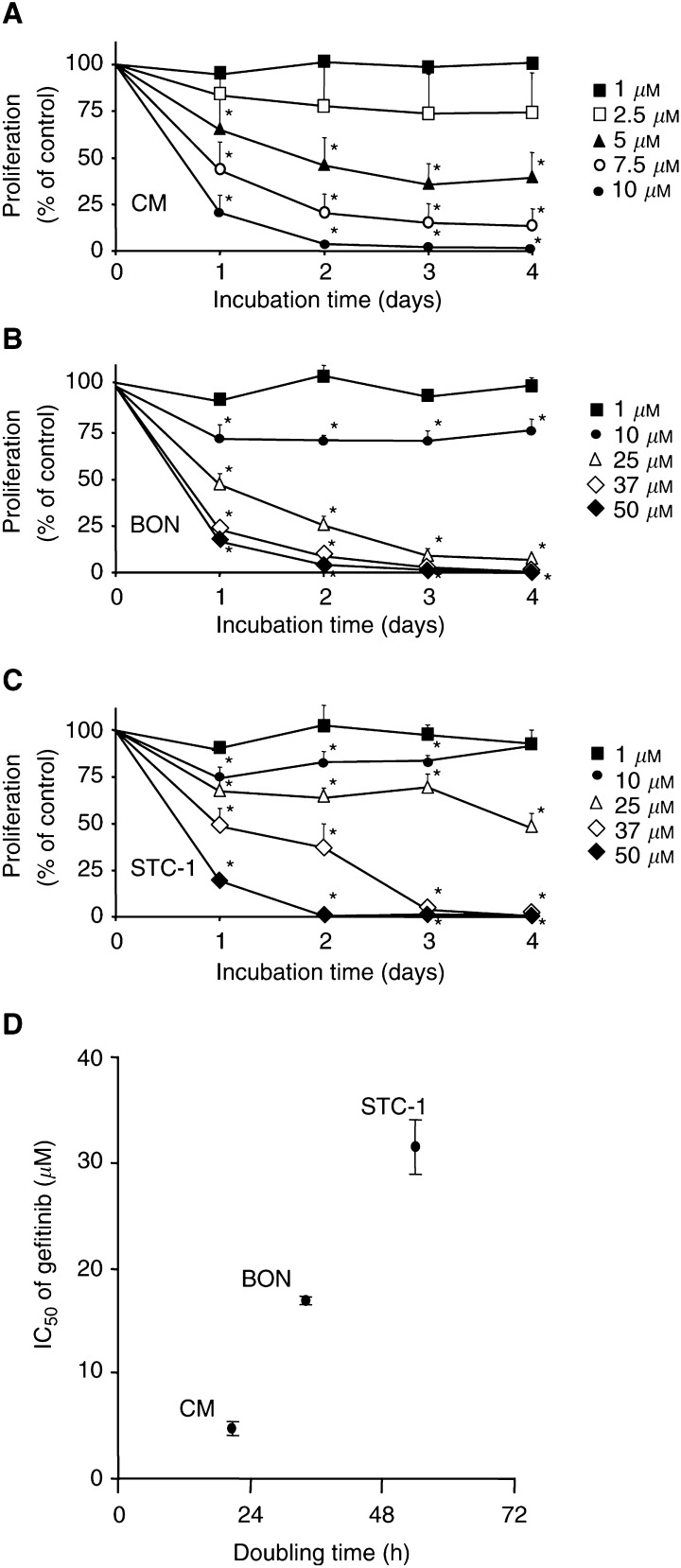
Gefitinib-induced growth inhibition. Gefitinib caused a time- and dose-dependent growth inhibition as measured by crystal violet staining. The IC_50_ value was 4.7±0.6 *μ*M in fast-growing CM cells (**A**), 16.8±0.4 *μ*M in moderate-growing BON cells (**B**), and 31.5±2.5 *μ*M in slow-growing STC-1 cells (**C**). (**D**) Correlation between the doubling time of NE gastrointestinal tumour cells and their sensitivity to gefitinib treatment. Data are given as the percentage of untreated controls (means±s.e.m. of four to five independent experiments). ^*^ Statistical significance (*P*<0.05).

Changes in the cell number caused by EGFR-TK inhibition were studied by performing crystal violet assays. Gefitinib (0–50 *μ*M) time- and dose-dependently inhibited the growth of all cell lines investigated (Figure 2A–C

). After 96 h of incubation, a decrease of almost 100% was observed. However, the IC_50_ values of gefitinib, determined after 48 h, differed between the three cell lines. While in fast-growing CM cells (doubling time: 21±1 h) the IC_50_ value of gefitinib amounted to 4.7±0.6 *μ*M, it was 16.8±0.4 *μ*M in the moderate-growing BON cells (doubling time: 34±4 h), but as high as 31.5±2.5 *μ*M in the slow-growing STC-1 cells (doubling time: 54±6 h) (Figure 2D).

Recently, we showed that nonradiolabelled MIBG specifically inhibited the growth of norepinephrine transporter (NET)-positive NE gastrointestinal tumour cells with an IC_50_ value of 7.8 *μ*M (Höpfner *et al*, 2002). Thus, it was intriguing to evaluate the possible synergistic antiproliferative effects of the combination of gefitinib with MIBG. Incubating NET-positive STC-1 cells for 3 days with combinations of sub-IC_50_ concentrations of MIBG (5 *μ*M) and gefitinib (10 *μ*M) resulted in an overadditive growth-inhibitory effect. While each drug alone decreased the growth of STC-1 cells by either 23% (10 *μ*M gefitinib) or 28% (5 *μ*M MIBG), the combination of both drugs led to a synergistic antiproliferative effect of more than 90% growth inhibition (data not shown).

### Gefitinib and cell-cycle regulation

**Figure 3:**
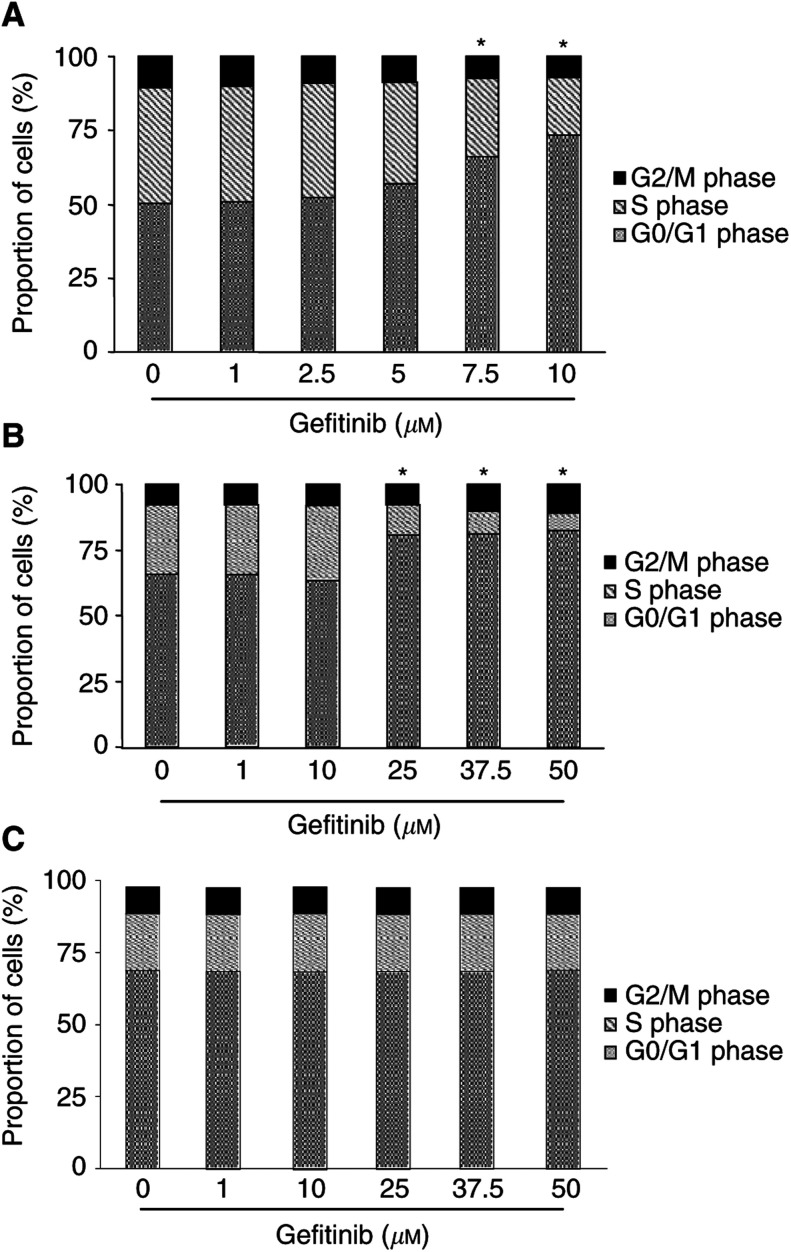
Induction of cell-cycle arrest by gefitinib. After 48 h of incubation with gefitinib, CM cells (**A**) and BON cells (**B**) dose-dependently accumulated in the G0/G1-phase of the cell cycle. Accordingly, the proportion of cells in the S and G2/M phases decreased in either cell line. In contrast, no significant changes of cell-cycle phases were observed in STC-1 cells (**C**). Means±s.e.m. of four independent experiments for each cell line are shown. The difference of the proportion of cells in a particular phase of the cell cycle *vs* control was significant for 7.5–10 *μ*M gefitinib in CM cells and for 10–50 *μ*M gefitinib in BON cells. ^*^ Statistical significance (*P*<0.05).

To test whether cell-cycle-arresting effects contributed to the antiproliferative potency of gefitinib in NE gastrointestinal tumour cells, we performed flow cytometric cell-cycle analysis. Challenging CM and BON cells with rising concentrations of gefitinib (0–10 *μ*M in CM, and 0–50 *μ*M in BON cells) for 48 h dose-dependently arrested CM and BON cells in the G1/G0 phase of the cell cycle, thereby decreasing the proportion of cells in the S phase and G2/M phase (Figure 3A, B

). Interestingly, gefitinib did not affect the cell cycle of STC-1 cells, even at the highest concentration of 50 *μ*M (Figure 3C).

### Proapoptotic effects of gefitinib

To check whether the induction of programmed cell death contributed to the antineoplastic effects of gefitinib, we investigated gefitinib-induced caspase-3 activation and DNA fragmentation in CM, BON and STC-1 cells.

**Figure 4:**
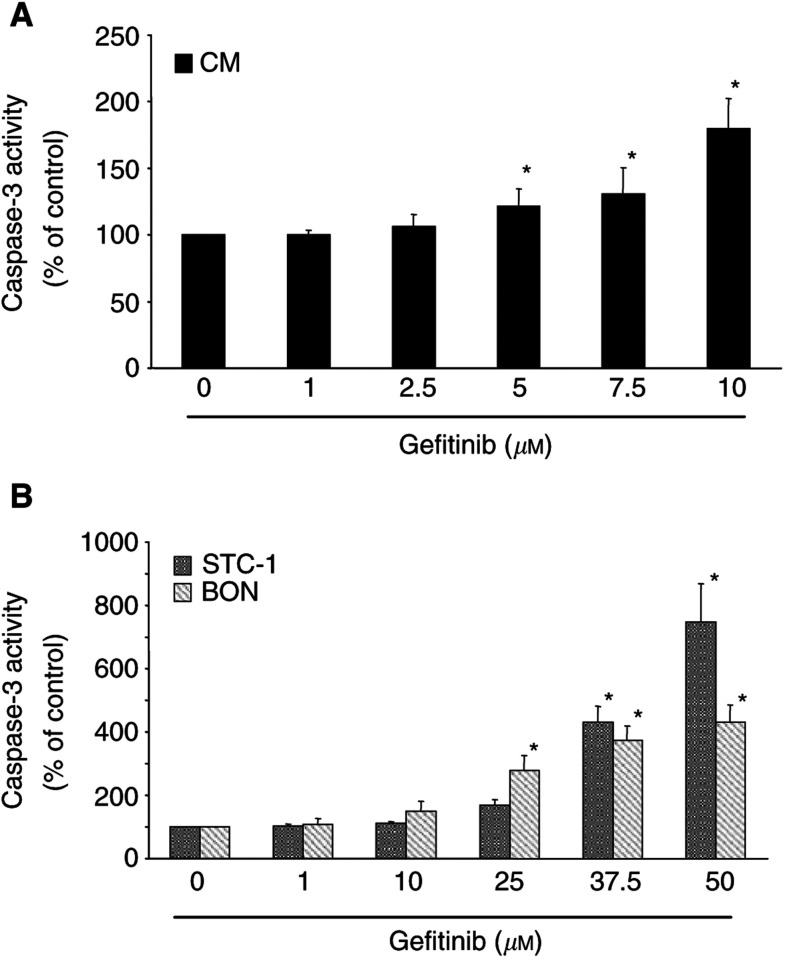
Gefitinib-induced caspase-3 activation. (**A**) In fast-growing CM cells, gefitinib (1–10 *μ*M) dose-dependently induced caspase-3 activation after 24 h of incubation. (**B**) Both the moderate-growing BON cells and the slow-growing STC-1 cells showed increases in caspase-3 activation when challenged with 1–50 *μ*M gefitinib. Referring to the respective IC_50_ values of gefitinib-induced growth inhibition, the apoptotic response in BON or STC-1 cells was even more pronounced than in CM cells. Data are given as the percentage of untreated control (means±s.e.m. of four independent experiments for each cell line). ^*^ Statistical significance (*P*<0.05).

Caspase-3 is a key enzyme in the apoptotic signalling pathway. In all the three cell lines, gefitinib induced a dose-dependent increase in caspase-3 activity (Figure 4

). The extent of caspase-3 activation differed between the three cell lines. Interestingly, caspase-3 activation was most pronounced in the slow-growing STC-1 cells (746±122% increase), which on the other hand had not displayed cell-cycle alterations in response to gefitinib treatment. This suggests that the antiproliferative effect of gefitinib in STC-1 cells is mainly caused by an induction of apoptosis. On the other hand, fast-growing CM cells, which exhibited the most pronounced G0/G1-arrest upon gefitinib treatment, showed the lowest, albeit still distinctive, caspase-3 activation of the three NE cell lines studied.

**Figure 5:**
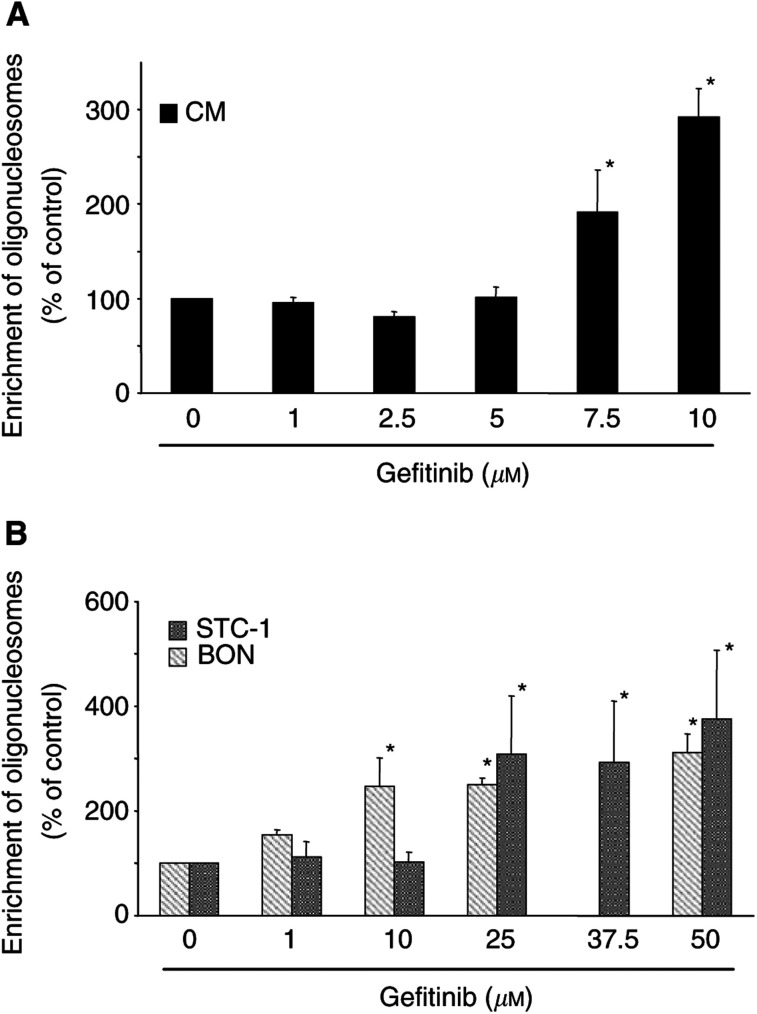
Gefitinib-induced DNA fragmentation. (**A**) Incubation of CM cells with 1–10 *μ*M gefitinib for 48 h led to a dose-dependent formation of apoptosis-specific oligonucleosomes. (**B**) Dose-dependent increases in DNA fragmentation of BON and STC-1 cells after 48 h incubation with gefitinib (1–50 *μ*M). Data are given as the percentage of untreated control (means±s.e.m. of four independent experiments for each cell line). ^*^ Statistical significance (*P*<0.05).

The fragmentation of DNA into mono- and oligonucleosomes is a hallmark of apoptosis. Gefitinib dose-dependently induced DNA fragmentation in NE gastrointestinal tumour cells (Figure 5

). After 48 h of incubation, an increase in DNA fragmentation of up to approx. 380% of the control values was observed. The results on DNA fragmentation confirm the notion that gefitinib potently induces apoptosis in NE gastrointestinal tumour cells. The extent of DNA fragmentation corresponded to the findings of caspase-3 activation in the respective cell line. Thus, strongest DNA fragmentation was seen in STC-1 cells, while CM cells showed less pronounced DNA fragmentation after incubation with gefitinib.

Since the antiproliferative effect of MIBG on NET-positive STC-1 cells was mainly caused by an induction of apoptosis (Höpfner *et al*, 2002), we wondered whether the overadditive antiproliferative effect of MIBG *plus* gefitinib was due to a synergistic induction of apoptosis. After 48 h of incubation, DNA fragmentation induced by either drug amounted to 116% (10 *μ*M gefitinib) or 105% (1 *μ*M MIBG), respectively. Combined incubation of STC-1 cells with MIBG (1 *μ*M) and gefitinib (10 *μ*M) revealed an overadditive activation of DNA-fragmentation of 141%.

### Gefitinib-induced differential gene expression

The present work demonstrates that gefitinib induces both apoptosis and cell-cycle arrest in NE gastrointestinal tumour cells. To study the involved cell-cycle and apoptosis genes at the transcriptional level, we performed cDNA microarray analysis of gefitinib-treated CM cells. For microarray experiments, we chose a concentration of 10 *μ*M gefitinib and a 48 h incubation time, which was shown to induce apoptosis and cell-cycle arrest in CM cells maximally.

We found 35 genes to be differentially expressed in CM cells upon gefitinib treatment. An asymmetric distribution of up- *vs* downregulated genes became apparent (see Table 2

**Table 2:** Transcripts differentially regulated in CM cells in response to gefitinib

**GenBank ID**	**Gene name**	**Function**	**Ratio**
U28014	*caspase 4*	Caspase	4.13
M63167	*rac* protein kinase alpha	Death kinase	3.27
S78085	Programmed cell death 2	Apoptosis-associated protein	3.13
U66879	*BCL2-antagonist of cell death (BAD)*	bcl-2 family protein	2.81
Y00285	Insulin-like growth factor 2 receptor	Growth factor receptor	2.55
U91985	DNA fragmentation factor alpha	DNA fragmentation protein	2.52
M35410	Insulin-like growth-factor binding protein 2	Growth factor-binding protein	2.41
AF022385	Programmed cell death 10	Apoptosis-associated protein	2.40
X59798	Cyclin D1	Cyclin	2.27
M81934	Cell division cycle 25B	Cell cycle protein	2.22
M32315	Tumour necrosis factor receptor superfamily, member 1B	Death receptor	2.03
U66469	Cell growth regulatory with ring finger domain	Cell cycle protein	1.99
U76376	harakiri, BCL2-interacting protein	bcl-2 family protein	1.83
M25753	Cyclin B1	Cyclin	1.81
X66363	PCTAIRE protein kinase 1	cdc2-related protein kinase	1.76
U25265	Mitogen-activated protein kinase kinase 5	Intracellular kinase network member	1.76
S40706	GADD153	Apoptosis-associated protein	1.72
U78798	TNF receptor-associated factor 6	Death receptor-associated protein	1.72
X85134	Retinoblastoma-binding protein 5	Cell cycle protein	1.71
L25080	*ras* homologue gene family, member A	Oncogene	1.70
M13228	v-*myc* myelocytomatosis viral-related oncogene	Oncogene	1.68
M33294	Tumour necrosis factor receptor superfamily, member 1A	Death receptor	1.67
M15796	Proliferating cell nuclear antigen	DNA replication	0.65
D21090	RAD23 homologue B	DNA damage signalling protein	0.65
S72008	Cell division cycle 10	Cell cycle protein	0.63
U39657	Mitogen-activated protein kinase kinase 6	Intracellular kinase network member	0.63
X66362	PCTAIRE protein kinase 3	cdc2-related protein kinase	0.61
U11791	Cyclin H	Cyclin	0.58
X60188	Extracellular signal-regulated kinase 1 (ERK1)	Cell cycle-regulating kinase	0.55
L05624	Mitogen-activated protein kinase kinase 1	Intracellular kinase network member	0.55
U75285	Survivin	Apoptosis-associated protein	0.54
X79389	Glutathione *S*-transferase theta 1	Xenobiotic transporter	0.54
U78876	Mitogen-activated protein kinase kinase kinase 3	Intracellular kinase network member	0.50
U21092	TNF receptor-associated factor 3	Death receptor-associated protein	0.47
L07414	Tumour necrosis factor superfamily, member 5	Death receptor ligand	0.42
M73812	Cyclin E1	Cyclin	0.26

). Among the genes known to be involved in the apoptosis signalling cascade, the expressions of *caspase 4, PDCD 2, BCL-2-antagonist of cell death (BAD)* and the bcl-2 family protein *harakiri* were overexpressed in gefitinib-treated cells. *GADD 153*, which plays an important role both in apoptosis and G0/G1 arrest, was also upregulated. Among the genes being downregulated, the cell-cycle regulating kinases *ERK1, CDC-like kinase 3, cyclin-dependent kinase 10* and the DNA-replication factor proliferating cell nuclear antigen *(PCNA)* were found.

### Gefitinib-mediated phosphorylation of ERK1/2 and p38 MAPK

**Figure 6:**
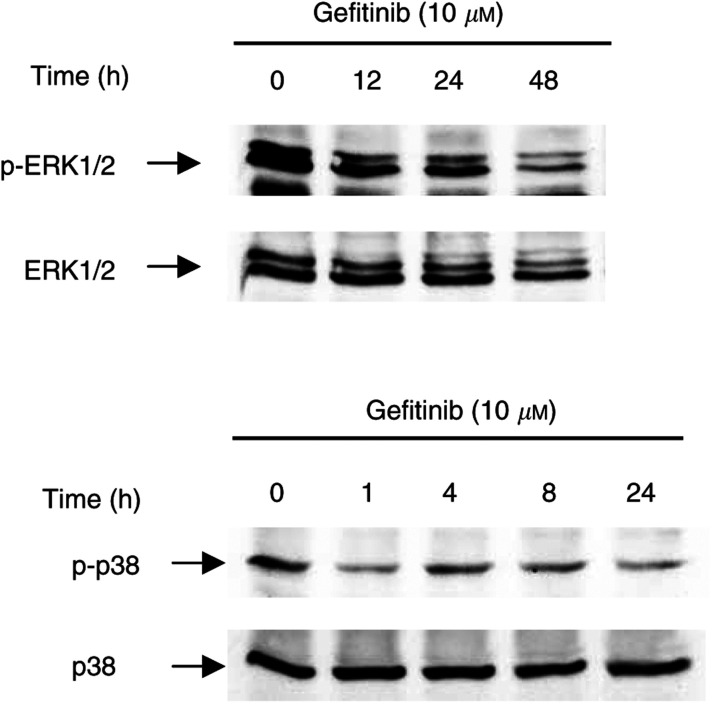
Effect of gefitinib on MAP kinase activity in CM cells. CM cells were treated with 10 *μ*M gefitinib for 0–48 h. At the indicated time points, the amount of phosphorylation of ERK1/2 and p38MAP kinases was analysed by Western blotting. (**A**) Gefitinib induced a time-dependent decrease in phosphorylated ERK1/2. (**B**) By contrast, no change in p38 MAPK phosphorylation was observed upon treatment with gefitinib.

To shed light on the signalling pathways influenced by EGFR-TK inhibition, we investigated time-dependent alterations of the phosphorylation of ERK1/2 and p38 MAPK, which are members of the MAPK family known to be involved in EGFR signalling in non-NE tumours. Incubating CM cells for 0–48 h with 10 *μ*M gefitinib revealed a time-dependent decrease in the phosphorylation of mitogenic ERK1/2 (Figure 6A

). In contrast, no significant alteration in the phosphorylation of the stress-activated p38 MAPK was observed (Figure 6B).

## DISCUSSION

EGFR signalling impacts on many aspects of tumour biology. The activation of EGFR has been shown to enhance tumour growth, invasion and spreading, and to inhibit apoptosis (Moghal and Sternberg, 1999; Wells, 2000). In addition, the expression of EGFR in tumours has been correlated with disease progression, poor survival, poor response to therapy (Brabender *et al*, 2000) and the development of resistance to cytotoxic agents (Wosikowski *et al*, 1997; Meyers *et al*, 1998). Thus, specific inhibition of EGFR and its intrinsic TK activity by different strategies (e.g. monoclonal antibodies, TK-inhibitors) has become a rationale for innovative cancer treatment (Ciardiello *et al*, 2000; Cullinane *et al*, 2000; Sirotnak *et al*, 2000; Overholser *et al*, 2000; Chan *et al*, 2001; Di Lorenzo *et al*, 2002; Sewell *et al*, 2002; Mendelsohn, 2003), – especially since the EGF receptor system is not only involved in tumour growth but also plays a significant role in tumour invasion, angiogenesis and adhesion (Woodburn, 1999; Ritter and Arteaga, 2003).

In the present study, we provide evidence that NE gastrointestinal tumour cells express both EGFR and the EGFR transactivating IGFR-*β*1 (Gilmore *et al*, 2002). Moreover, gefitinib, a specific EGFR-TK inhibitor, was found to inhibit potently the growth of NE gastrointestinal tumour cells by inducing cell-cycle arrest and/or apoptosis. The inhibition of EGFR-TK by gefitinib led to a time- and dose-dependent growth inhibition by almost 100%. The effects were observed under foetal calf- and/or horse serum-containing conditions, that is, in the presence of growth factors like EGF, IGF and TGF-*α*. Under these *in vivo-like* serum conditions, gefitinib was very potent, and thus qualifies as a promising novel drug to be tested clinically in metastatic NE gastrointestinal tumour disease.

The growth pattern of NE gastrointestinal tumours exhibits an astonishingly wide spectrum ranging from very slow- to moderate-growing types and finally to fast-growing, very aggressive types of tumours (Öberg, 1994). In view of this particular background, it was important to check whether gefitinib was similarly capable of inhibiting the proliferation of NE tumours with different growth characteristics and to study the involved signalling pathways. Therefore, we chose three models of NE gastrointestinal tumour disease, each characterised by a different growth pattern and different origin: first, human insulinoma CM cells, which have a doubling time of 21 h and represent ‘fast-growing’ NE tumour cells. Second, human pancreatic carcinoid BON cells with a doubling time of 34 h, which represent ‘moderate-growing’ NE tumour cells. Third, ‘slow-growing’ NE tumour cells of the gut (STC-1 cells) with a doubling time of 54 h. Each cell line was cultured under its optimal growth conditions, which resulted in different serum concentrations among the three cell lines. Nevertheless, the different serum concentrations were not responsible for the different antineoplastic efficacies of gefitinib observed in the three cell lines (data not shown). Although gefitinib effectively inhibited tumour growth in each model, differences concerning dose–response relationships and involved signalling pathways became apparent. In fast-growing CM cells that displayed the highest sensitivity to gefitinib (IC_50_ of 4.7±0.6 *μ*M), the antineoplastic effect was based on both a moderate induction of apoptosis and a strong G1/G0 phase arrest of the cell-cycle. In moderate-growing BON cells also an induction of apoptosis as well as an arrest in the G1/G0 phase of the cell-cycle were observed. However, the concentrations needed to induce the antineoplastic effects were significantly higher (IC_50_ of 6.8±0.4 *μ*M), and the apoptotic response was more pronounced than in CM cells. On the other hand, in slow-growing STC-1 cells displaying the lowest sensitivity to gefitinib (IC_50_ 31.5±2.5 *μ*M), the antineoplastic effect was based on a strong induction of apoptosis, while no significant cell-cycle alterations were observed.

The shift from a more cell-cycle arrest related action in fast-growing NE tumour cells to an (almost) exclusively apoptosis-related effect in slow-growing NE tumour cells may be important to be taken into account when considering novel combination therapies. In this respect, we were interested in the antiproliferative synergism between gefitinib and the norepinephrine derivative MIBG. Nonradiolabelled MIBG has recently been suggested as an innovative drug for the treatment of NE gastrointestinal tumours expressing plasma membraneous norepinephrine transporters (NET) (Taal *et al*, 1996; Höpfner *et al*, 2002). Since the coapplication of MIBG and gefitinib resulted in an overadditive, synergistic antiproliferative action in STC-1 cells, it is intriguing to speculate whether in NET-positive NE tumours, the combination of MIBG and gefitinib might be superior to either drug alone. The mechanisms underlying the observed synergism, however, need further investigation.

To further characterise the antiproliferative action of gefitinib in NE gastrointestinal tumour cells, we studied the cell-cycle phase, at which gefitinib might act. Upon gefitinib treatment, the proportion of cells in the G1/G0 phase significantly increased both in CM and BON cells. This suggests that gefitinib acts at the G1/S checkpoint. Cell-cycle arrest by gefitinib at the G1/S checkpoint had previously been described for other tumours, such as head and neck cancer (Huang *et al*, 2002), malignant pleural mesothelioma (Jänne *et al*, 2002) and breast cancer (Janmaat *et al*, 2002). The latter tumour types mainly represent fast-growing cancers known to be very sensitive to growth factors. Many of these cancers produce and release growth factors and thereby autostimulate themselves. This has also been suggested for NE gastrointestinal tumours in which self-produced transforming growth factor *α* (TGF-*α*) and insulin-like growth factor (IGF) may autostimulate or transactivate EGFR and thereby promote tumour cell growth (Nilsson *et al*, 1993, 1995). As stromal components of midgut carcinoids also express EGFR, growth factors like TGF-*α* may similarly contribute to the desmoplastic reaction typical of midgut carcinoids (Öberg, 1994). Thus, EGFR-TK inhibition might as well be a worthwhile strategy to pursue for the control of the desmoplastic reaction and fibrosis of carcinoid disease.

Induction of apoptosis by gefitinib has been reported previously (Ciardiello *et al*, 2000; Huang *et al*, 2002; Janmaat *et al*, 2002). However, the underlying mechanisms are not yet well understood (Mendelsohn, 2002). In this paper, we report on the activation of the proapoptotic enzyme caspase-3, which preceded the fragmentation of nuclear DNA into oligonucleosomes. This indicates that in NE tumour cells, gefitinib-mediated apoptosis is caspase-3 dependent. To support our functional data, we additionally performed cancer-specific cDNA arrays, spotted with genes related to proliferation, apoptosis and cell cycle. Treatment with gefitinib resulted in an upregulation of proapoptotic and a suppression of antiapoptotic genes of NE gastrointestinal tumour cells (Table 2). The changes in the expression pattern of the several apoptosis-related genes were consistent with both our functional data, and reports showing that suppression of antiapoptotic and overexpression of proapoptotic proteins caused programmed cell death (Zamzami *et al*, 1996). Besides an overexpression of the cell cycle and apoptosis relevant *GADD153* gene (see below), a strong upregulation of *caspase 4*, which has been implicated to play an important role in the execution of apoptosis (Kamada *et al*, 1997), was observed. We also observed a strong induction of proapoptotic *BAD* at the transcriptional level, suggesting a potential involvement of this protein in gefitinib-induced apoptosis. As suggested by others, a dephosphorylation of Akt/PKB by gefitinib may be responsible for the activation of proapoptotic *BAD* (Albanell *et al*, 2001). However, further investigations will have to clarify the exact role of *BAD* in gefitinib-induced apoptosis of NE tumour cells.

Gefitinib's mode of action is known to include the suppression of EGFR-TK-induced activation of MAP kinases, such as ERK1/2 (Albanell *et al*, 2001; Mendelsohn, 2002). Confirming this notion, gefitinib did cause a time-dependent dephosphorylation and transcriptional downregulation of ERK1/2 in NE tumour cells. In addition, we observed an upregulation of the growth arrest and DNA-damage inducible gene, GADD153, which is involved in G1/G0 arrest (Wang and Ron, 1996; Oh-Hashi *et al*, 2001). The expression of GADD153 is known to be regulated by both ERK1/2 and p38 MAPK: While p38 MAPK stimulates the expression of GADD153, ERK1/2 inhibits the expression (Kultz *et al*, 1998; Oh-Hashi *et al*, 2001). Interestingly, we did not observe any significant effect of gefitinib on p38 MAPK activity in NE tumour cells. This suggests that the upregulation of GADD153 by gefitinib is mediated by a downregulation of ERK1/2, but not by increased p38 MAPK activity.

Although gefitinib's antiproliferative effect results from the specific inhibition of EGFR-sensitive TK activity, gefitinib's antineoplastic potency not simply reflects the number of EGFRs expressed. While some studies have reported a positive correlation between EGFR expression and gefitinib's antineo-plastic action (Meye *et al*, 2001; Normanno *et al*, 2002), others have found no such relationship (Ciardiello *et al*, 2000; Sirotnak *et al*, 2000). One possible explanation for a missing correlation between EGFR expression and gefitinib's antiproliferative activity may be the presence of constitutively active EGFR mutations, such as EGFRvIII (Moscatello *et al*, 1995; Wikstrand *et al*, 1995; Nishikawa *et al*, 1994). However, in neither NE gastrointestinal tumour model used in this study, an expression of EGFRvIII mutation could be detected and hence could not contribute to the differing effects of gefitinib. Another reason for a missing positive correlation could be EGFR transactivation by other growth factor receptors (Arteaga, 2002). In this respect, Gilmore *et al* (2002) recently demonstrated a transactivation of EGFR-TK by the IGFR. Gefitinib suppressed the EGFR transactivation by IGFR, and thereby induced apoptosis in breast cancer cells. EGFR transactivation may also be important in NE gastrointestinal tumours in which IGFR is commonly expressed and has been implicated in growth control (Nilsson *et al*, 1993; Wulbrand *et al*, 2000).

To conclude, our study provides evidence that the EGFR-TK inhibitor gefitinib induces both cell-cycle arrest and apoptosis in NE gastrointestinal tumour cells. Thus, gefitinib represents a promising novel drug to be tested in patients with metastatic NE gastrointestinal tumour disease.
